# “Top-down bottom-up” estimation of per capita cost of new-born care interventions in four regions of Ghana: beyond implementation to scalability and sustainability

**DOI:** 10.1186/s13561-021-00307-1

**Published:** 2021-02-26

**Authors:** Robert Kaba Alhassan, Edward Nketiah-Amponsah, Nana A. Y. Twum-Danso, John Bawa, Williams Kwarah, Sebnem Ucer, Abdul Fatawu Ibn Abass

**Affiliations:** 1 Centre for Health Policy and Implementation Research, Institute of Health Research, University of Health and Allied Sciences, PMB 31, Ho, Volta Region Ghana; 2 Department of Economics, University of Ghana, Legon, Ghana; 3 TD Health, Accra, Ghana; 4 PATH, Accra, Ghana; 5 Kybele, NC 27023 Lewisville, USA

**Keywords:** Top-down, Bottom-up, New-born care, Marking every baby count initiative, Ghana, Per capita cost, Evaluation, Health policy, Scalability, Sustainability

## Abstract

**Background:**

Limited financial, human and material health resources coupled with increasing demand for new-born care services require efficiency in health systems to maximize the available sources for improved health outcomes. Making Every Baby Count Initiative (MEBCI) implemented by local and international partners in 2013 in Ghana aimed at attaining neonatal mortality of 21 per 1000 livebirths by 2018 in four administrative regions in Ghana. MEBCI interventions benefited 4027 health providers, out of which 3453 (86%) were clinical healthcare staff.

**Objective:**

Determine the per capita cost of the MEBCI interventions towards enhancing new-born care best practices through capacity trainings for frontline clinical and non-clinical staff.

**Methods:**

Parameters for determining per capita cost of the new-born care interventions were estimated using expenditure on trainings, supervisions, monitoring and evaluation, advocacy, administrative/services and medical logistics. Data collection started in October 2017 and ended in September 2018. Data sources for the per capita cost estimations were invoices, expense reports and ledger books at the national, regional and district levels of the health system.

**Results:**

Total of 4027 healthcare providers benefited from the MEBCI training activities comprising of 3453 clinical staff and 574 non-clinical personnel. Cumulative cost of implementing the MEBCI interventions did not necessarily match the cost per capita in staff capacity building; average cost per capita for all staff (clinical and non-clinical staff) was approximately US$ 982 compared to a per capita cost of US$ 799 for training only core clinical staff. Average cost per capita for all regions was approximately US$ 965 for all staff compared to US$ 777 per capita cost for only clinical staff. Per capita cost of training was relatively lower in regions with more staff than regions with lower numbers, perhaps due to economies of scale.

**Conclusion:**

The MEBCI intervention had a wide coverage in terms of training for frontline healthcare providers albeit the associated cost may be potentially unsustainable for Ghana’s health system. Emerging digital training platforms could be leveraged to reduce per capita cost of training. Large-scale on-site batch-training approach could also be replaced with facility-based workshops using training of trainers (TOTs) approach to promote efficiency.

## Background

According to Ghana Demographic and Health Survey (GDHS) report [1], Ghana has over the years recorded significant improvements in infant and under-five mortality rates with infant mortality rate per 1000 population reducing from 77 in 1988 to 41 in 2014; likewise, under-five mortality rate has improved from 155 per 1000 population in 1988 to 60 per 1000 population in 2014. However, the country continues to battle with challenges of new-born care. In 2015 Ghana recorded a neonatal mortality rate of 28 per 1000 live births, marginally above the African average of 28 [2].

According to a WHO report [2], for Ghana to attain the 2030 target of 12 neonatal deaths per 1000 live births, there is the need to invest in more cost-effective and efficient health interventions to promote greater impact and sustainability of these public health interventions. The quest for greater efficiency in the execution of health care interventions is particularly compelling for resource-poor countries such as Ghana since it is critical to meeting the increasing demand for basic health care services in the midst of limited financial, human and material health resources [3–7].

While health care for the new-born remains a priority area for the government of Ghana, programmes targeting these vulnerable populations must work within a constrained resource envelope, especially as donor budgetary support for the health sector is in a declining trajectory [8]. As part of efforts to support the Government of Ghana (GoG) to attain the goal of reducing neonatal mortality, the Ministry of Health, Ghana Health Service and other local and international partners initiated a joint collaborative project in 2013 called Making Every Baby Count Initiative (MEBCI). The project aimed at attaining neonatal mortality of 21 per 1000 livebirths by 2018 in four administrative regions in Ghana (names withheld for anonymity).

The overall goal of MEBCI intervention is that by 2018, 90% of new-borns delivered in selected healthcare facilities in target intervention regions will receive essential new-born care and appropriate interventions to address asphyxia, infection, and prematurity as per GoG standard guidelines for new-born care [9]. Moreover, the MEBCI intervention sought to complement efforts towards establishing mechanisms to sustain impact at the national level and in the four target regions.

The MEBCI interventions entailed initial health facility assessments prior to start of staff trainings to establish facility capacity for new-born care; clinical skills training for care givers in the form of Essential Care for Every Baby (ECEB), Helping Babies Breathe (HBB), Infection Prevention (IP), Kangaroo Mother Care (KMC), and follow-up visits. The remaining components of the MEBCI interventions included advocacy and policy dialogues, and related administrative/support activities on the project deliverables. This study conducted a top-down bottom-up per capita cost estimations of new-born care interventions implemented in four administrative regions in Ghana.

## Methods

### Design

Unit cost calculations were used to estimate the per capita cost of MEBCI interventions along the spectra of inputs and outputs. Proxies for the input factors were the direct and indirect cost of the MEBCI interventions quantified in absolute Ghana Cedis (GHC) and United States Dollars (US$) equivalence. Output indicators were proxied by the average training scores of MEBCI-trainees on HBB and ECEB. Other output indicators were total number of staff trained in HBB and ECEB; neonatal asphyxia cases; premature cases; neonatal hypothermia; neonatal sepsis, and still births throughout the period of the MEBCI interventions.

The cost analysis technique was retrospective in nature where administrative records were audited alongside desk review of financial expenditure records at the various cost centers involved in the MEBCI intervention implementation at the national, regional and district levels.

### Cost evaluation sites

The evaluation was done in four (4) out of the then ten (10) administrative regions of Ghana. Since the project was implemented before the creation of the new administrative regions, this paper makes reference to the old regional demarcations. Total population of healthcare facilities engaged in MEBCI were 155, from the four intervention regions. These include 4 regional hospitals, 99 district hospitals, 4 polyclinics and 48 health centers (see Table 1). Cost data were retrieved from the local funding agent in Accra; the four Regional Health Directorates (RHDs), and Regional Hospitals (RHs) in the target regions.

**Table 1:** Health facilities engaged in MEBCI interventions: disaggregated by region

Health Facilities	Region A	Region B	Region C	Region D	Total
**Regional Hospitals**^**1**^	**1**	**1**	**1**	**1**	**4**
**District Hospitals**	**36**	**19**	**24**	**20**	**99**
**Polyclinics**	**0**	**2**	**2**	**0**	**4**
**Health Centers**	**20**	**8**	**10**	**10**	**48**
**Total**	**57**	**30**	**37**	**31**	**155**

Source: Local Funding agent administrative data (2017)

Legend: Note: names of regions anonymized with letters (A, B, C & D)

### Sampling

All available data on financial cost pertaining to MEBCI activities at the national, regional and district levels. All four intervention regions and their respective regional hospitals were evaluated in terms of expenditure reports on the MEBCI interventions.

Additionally, an audit census was done on all cost data in the four RHDs and regional hospital which were responsible for budgeting, disbursing and expending on provider training activities. Likewise, all expenditure records at the local funding agent in Accra were retrieved and analyzed to determine global cost of the interventions in terms of advocacy, developing training curricula, building capacity and national leadership in new-born care.

### Inclusion and exclusion criteria

Inclusion criteria for the cost data review were: the region and its respective regional hospital should have benefited from MEBCI intervention activities, and beneficiary health facilities and staff should have received MEBCI provider trainings and follow-up visits. Regional hospitals that did not received the core MEBCI intervention were excluded. Likewise, tertiary level facilities were excluded from the cost estimations since they were not part of the MEBCI interventions design.

### Data collection/exploration

**Fig. 1:**
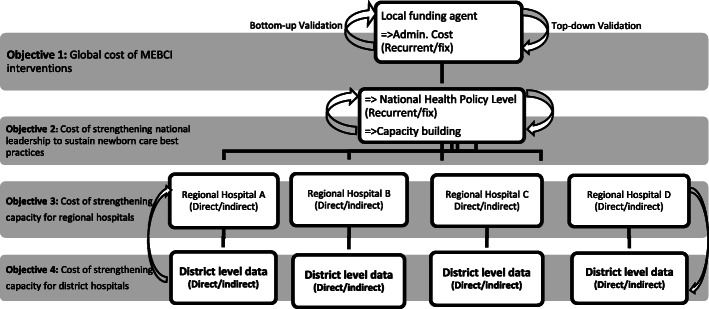
Top-down bottom-up procedure for data collection and cost efficiency analysis

A “top-down bottom-up” approach was used in the cost data collection process and estimation of the per capita cost of the MEBCI interventions. The MEBCI interventions lasted for five (5) years (September 2013 to August 2018) with funding from an international donor, in the United Kingdom (UK) and implemented jointly by local government agencies within the health sector. Data were first retrieved from the level of the intervention’s funder, in a top-down approach. The bottom-up approach entailed data collection from direct level beneficiaries of the MEBCI Project, namely the national health policy level, the four RHDs (which directly administered funds for the district hospitals, polyclinics and health centers) and the four regional hospitals. Figure 1 shows the framework for data collection and the cost analysis.

### Data sources

Data were largely retrieved from administrative records at the local funding agent level and local implementing partners. Data included administrative and cost accounting records. Other data sources were annual budgets, invoices, expense reports and ledger books by the various cost centers.

Key informants during the data collection at funding agent were the project administrator and accountant. At the RHDs, the key informants were mostly MEBCI regional focal persons, regional accountants or their delegates. Expenditure items reported included trainings, supervisions, monitoring and evaluation, advocacy, support/administrative running costs and logistics. Provider training data were used to estimate average staff time lost to MEBCI trainings. These person-hours were costed per capita in monetary terms with the aim of deriving opportunity cost of the MEBCI interventions to the individual staff using a liberal parameter of the prevailing national daily minimum wage (NDMW) [10]. Opportunity cost was used as proxy for indirect cost of the new-born care interventions to health staff engaged throughout the period of the interventions.

### Data collection instruments

Four set of guideline instruments were designed and validated over a period of three months before implementation. The instruments were uniquely designed to collect cost data at all cost centres. Each cost analysis tool had sections, unit cost, quantities and frequency of cost, and total cost per item or activity as applicable. Specific cost items were further disaggregated into direct (fixed and recurrent) and indirect (fixed and recurrent) costs.

### Data collection process

A team of two data collectors with academic backgrounds in Health Economics were recruited and trained to collect data. Data collection at funding agent level started from 23rd October, 2017 and ended on 30th November, 2017. At the RHDs, data collection was done at different time intervals in the first quarter of 2018 while cost data from the regional and district levels were in the third quarter of 2018.

The field workers were directly supervised by the lead consultants for the cost analysis of the MEBCI Project. Double entry of data was done by the two trained evaluators prior to data coding and cleaning to minimize data entry errors and promote validity of the data. Field data were captured into Microsoft Excel (2013) and later exported to STATA (version 12.0) for analysis.

### Data completeness

Cost records retrieved from the funding agent spanned from September 2013 to August 2017 which represented the first four fiscal years of the five-year project. At the RHDs, data from each of the four regions were collected at different time periods as stated earlier. Data from national implementing agents were retrieved between June and September 2018. It was observed that cost data had different dates in the respective regions depending on the fiscal year when the MEBCI project was started in the pertinent region. Cost data accessed from the RHDs ranged from November 2014 to March 2018.

### Cost estimations and analysis

Cost figures were derived from reported lump sums of expenditures from all cost centres. Parameters for the cost estimation included direct cost and indirect cost, including quantified cost of one day of staff time lost to training (proxy for opportunity cost). The unit cost analysis for the MEBCI interventions was done based on “cost of MEBCI activity” per “year” per “region” and later decomposed into “cost per capita” (i.e. individual staff).

The unit of analysis for the provider training cost was the four regions and the MEBCI implementation periods (i.e. fiscal years). At the funding agent level, cost estimations were done based on cost related to regional and district levels. Cost expenditure data were categorized into fixed cost and recurrent cost. Fixed cost comprised of purchase of project vehicles, project office rent and sunk cost on equipment and sub-agreements with intervention regions. Recurrent cost components included staff salaries, international and local travels for staff of GHS and consultants, consultative/sub-committee meetings, advertisements, bills and utilities, stationery, repairs and maintenance.

## Results

### Cost estimation of new-born care interventions at national and regional levels

**Fig. 2:**
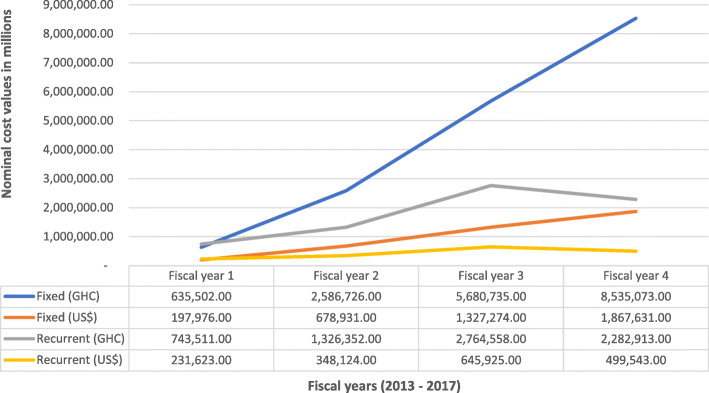
Cost at the funding agent level: disaggregated by fiscal year. Source: Cost per fiscal year funding agent data (Sept, 2013 – Aug, 2017); Note: There were no readily available uniform US$ equivalence of all recorded expenditures in the four regions hence, the US$ equivalence as quoted in this report are based on the current prevailing exchange rate of 1 US$ = 4.45 GHC. US$ 4.45 Source: www.oanda.com/currency/converter Accessed on 27/09/2018. Accessed on 24/09/2018

Cumulative amount of GH₵ 24,555,370.41 (US$ 5,518,061^1^) was recorded as expenditure for fixed and recurrent cost activities between September 2013 and August 2017. Out of this amount, fixed cost expenditure constituted 71% while recurrent cost represented 29% of the total expenditure. Cost per year of implementation of the MEBCI intervention showed that the first project year (fiscal year one) recorded the least expenditure of GH₵ 1,379,013.12 (equivalent to US$ 429,599.10)^2^ while the fourth project year (fiscal year four) recorded the highest cost with an expenditure of GH₵ 10,817,985.59 (equivalent to US$ 2,367,174.09)^3^ (see Fig. 2). It is however important to elucidate the point that these fix and recurrent costs alone do not make much meaning hence these cost figures were linked to the cost per capita of the interventions in terms of capacity building and related activities for staff to enhance new-born care.

**Fig. 3:**
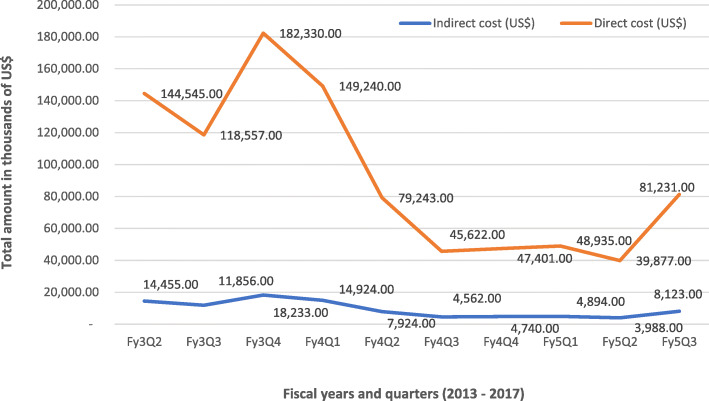
Cost of regional hospital related activities disaggregated by direct and indirect cost. Source: Regional Hospitals Data (2014–2018); Note: Data points for fiscal year1, year2 and Fiscal year 3 quarter 1 were not available in the cost data hence their exclusion in this graph. Legend: Fy3Q2 (1st Dec, 2015 – 29th Feb, 2016); Fy3Q3 (1st March, 2016 – May, 2016); Fy3Q4 (1st Jun, 2016 – 31st Aug, 2016); Fy4Q1 (1st Sept, 2016 – 31st Nov, 2016); Fy4Q2 (1ST Dec, 2016 – 28th Feb, 2017); Fy4Q3 (1st March, 2017 – 31st May, 2017); Fy4Q4 (1st Jun, 2017 – 31st Aug, 2017); Fy5Q1 (1st Sept, 2017 – 30th Nov, 2017); Fy5Q2 (1st Dec, 2017 – 28th Feb, 2018); Fy5Q3 (1st March, 2018 – 31st May, 2018); Note: There were no readily available uniform US$ equivalence of all recorded expenditures in the four regions hence, the US$ equivalence as quoted in this report are based on the current prevailing exchange rate of 1 US$ = 4.45 GHC. US$ 4.45 Source: www.oanda.com/currency/converter Accessed on 27/09/2018. Accessed on 24/09/2018

**Fig. 4:**
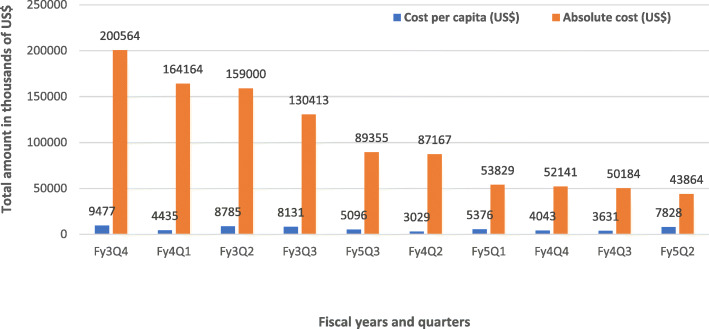
Cost of regional hospital related activities disaggregated by cost per capita and absolute cost. Source: Regional Hospitals Data (2014–2018); Note: Data points for fiscal year1, year2 and Fiscal year 3 quarter 1 were not available in the cost data hence their exclusion in this graph. Legend: Fy3Q2 (1st Dec, 2015 – 29th Feb, 2016); Fy3Q3 (1st March, 2016 – May, 2016); Fy3Q4 (1st Jun, 2016 – 31st Aug, 2016); Fy4Q1 (1st Sept, 2016 – 31st Nov, 2016); Fy4Q2 (1ST Dec, 2016 – 28th Feb, 2017); Fy4Q3 (1st March, 2017 – 31st May, 2017); Fy4Q4 (1st Jun, 2017 – 31st Aug, 2017); Fy5Q1 (1st Sept, 2017 – 30th Nov, 2017); Fy5Q2 (1st Dec, 2017 – 28th Feb, 2018); Fy5Q3 (1st March, 2018 – 31st May, 2018); Note: There were no readily available uniform US$ equivalence of all recorded expenditures in the four regions hence, the US$ equivalence as quoted in this report are based on the current prevailing exchange rate of 1 US$ = 4.45 GHC. US$ 4.45 Source: www.oanda.com/currency/converter Accessed on 27/09/2018. Accessed on 24/09/2018

As showed in Figs. 3 and 4, the cumulative cost of the new-born care at the national and regional levels are relatively high because activities at these levels were mostly recurrent and fix national and health policy direction activities coupled with capacity building activities. Activities at these aimed at strengthening national leadership was decomposed into cost per the domains of workshops, training- activities and development of guidelines on new-born standard and Kangaroo Mother Care (KMC) guidelines. The composite cost of strengthening capacity for the four beneficiary regional hospitals was also disaggregated into direct (cost directly related to the new-born care interventions) and indirect cost (cost not directly related the new-born care interventions but relevant to implementing the interventions) expenditures with the overall direct cost accounting for 91% of the total cost while indirect cost constituted 9%. Cost per capita in capacity building for at the regional hospital level showed that the highest per capita cost highest in third-year of the project while the fifth-year project year recorded the least per capita cost (see Fig. 4).

### Profile of beneficiary staff of the new-born care interventions

According to provider training records retrieved from the funding agent, a total of 4027 individuals were involved in the MEBCI training activities. This number includes key clinical staff (*n* = 3453) and non-clinical personnel (*n* = 574). For the purposes of this evaluation, only data related to key clinical staff were further explored and analyzed. Beneficiaries of the MEBCI training interventions at the district hospitals, polyclinics and health centres were generally youthful with a mean age of 32 years and average of 6 years of work experience. Out of the total number of 3453 clinical providers trained, 85% were females; midwives constituted 61% with the least being physician assistants (1%). Approximately 90% of the staff trained were from district hospitals while staff from polyclinics and health centres constituted 2 and 8% respectively. Two thirds of the total number of trained clinical staff were from public (government and quasi-government) facilities and the remaining from faith-based health facilities (see Table 2).

**Table 2:** Summary statistics on district providers trained and followed-up

Staff Characteristics	Mean	Std. Dev.
**Age (***n* **= 3367)**	**32**	**8.4**
**Years of work experience (***n* **= 3323)**	**6**	**7.7**
**HBB0 score (***n* **= 3450)**	**21**	**2.3**
**ECEB0 score (*n*** **= 3450)**	**27**	**2.4**
**Sex (*n*** **= 3453)**	**Freq.**	**%**
**Male**	**506**	**15**
**Female**	**2947**	**85**
**Cadre (*n*** **= 3453)**	**Freq.**	**%**
**Medical doctors**^a^	**165**	**5**
**Anesthetists**	**190**	**6**
**Nurses**^b^	**941**	**27**
**Midwives**	**2106**	**61**
**Physician assistants**	**51**	**1**
**Facility type (***n* **= 3449)**^c^	**Freq.**	**%**
**District hospital**	**3101**	**90**
**Polyclinic**	**59**	**2**
**Health center**	**289**	**8**
**Regions (*n*** **= 3453)**	**Freq.**	**%**
**Region A**	**1368**	**40**
**Region B**	**790**	**23**
**Region C**	**806**	**23**
**Region D**	**489**	**14**
**Ownership (*n*** **= 3453)**	**Freq.**	**%**
**Private**^d^	**1234**	**36**
**Public**^e^	**2219**	**64**

Source: MEBCI training data (2018)

Legend: Helping Babies Breathe First Assessment (HBB0, the first OSCE score immediately after the training) and Essential Care for Every Baby First Assessment (ECEB0, the first OSCE score immediately after the training)

^a^Includes specialist doctors such as pediatricians, obstetrics and gynecology

^b^Includes health assistants, perioperative nurses, enrolled nurses, pediatric nurses, community health nurses, critical and emergency care nurses, ward assistants

^c^One provider each was drawn from the four regional hospitals which constitutes less than 1% of the total number of providers trained

^d^Facilities owned by faith-based organizations

^e^Facilities owned by Ghana Health Service or Quasi-government organizations

### Cost per capita of capacity building for new-born care

**Fig. 5:**
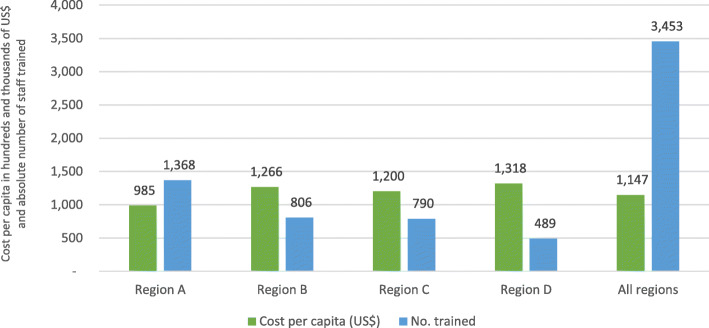
Number of district-level providers trained and the cost per capita in absolute and staffing category. Source: Regional health directorates (RHDs) in four MEBCI regions (Jan-March, 2018). NOTE: *Cost per capita estimations based on consolidated direct and indirect cost components; There were no readily available uniform US$ equivalence of all recorded expenditures in the four regions hence, the US$ equivalence as quoted in this report are based on the current prevailing exchange rate of 1 US$ = 4.45 GHC. US$ 4.45 Source: www.oanda.com/currency/converter Accessed on 27/09/2018. Accessed on 24/09/2018

**Fig. 6:**
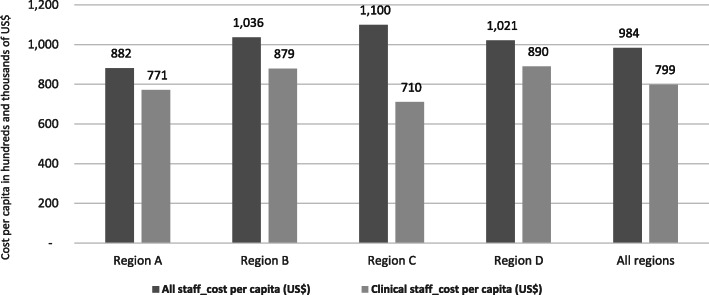
Number of district-level providers trained per capita: disaggregated by region. Source: Regional health directorates (RHDs) in four MEBCI regions (Jan-March, 2018). NOTE: *Cost per capita estimations based on consolidated direct and indirect cost components; Note: There were no readily available uniform US$ equivalence of all recorded expenditures in the four regions hence, the US$ equivalence as quoted in this report are based on the current prevailing exchange rate of 1 US$ = 4.45 GHC. US$ 4.45 Source: www.oanda.com/currency/converter Accessed on 27/09/2018. Accessed on 24/09/2018

It was observed that the cumulative cost of implementing the MEBCI interventions did not necessarily match the cost per capita in capacity building for new-born care. For instance, it was observed that the region that recorded the highest cost of training rather recorded the least in cost per capita (i.e. US$ 985 per training a staff). Additionally, for those trained to be followed up beyond the initial training, the regional distributions in cost per capita can be found in Fig. 4. The per capita cost for new-born care was further disaggregated into per capita cost for all categories of staff versus core clinical staff. After factoring in the indirect cost components of the capacity building activities, it was found that the average cost per capita for all staff (including non-clinical staff) was approximately US$ 982 compared to US$ 799 for the core clinical staff. Subsequently, the cost per capita was estimated using only the direct cost components of training. The records revealed that the average cost per capita for all regions was approximately US$ 965 for all cadre of staff (including non-clinical staff) compared to US$ 777 per capita cost for only clinical staff (see Figs. 5 & 6). Regions with higher number of trained staff recorded lower per capita cost of the new-born care interventions and vice versa, suggesting these regions perhaps enjoyed economies of scale and were more efficient in implementing the interventions implementation (see Figs. 5 & 6).

## Discussion

This evaluation examined the nominal and cost per capita of interventions that targeted the national, regional and district level stakeholders of new-born care in Ghana. Findings on the overall cost the new-born care interventions at these different levels of implementation raise important questions on the sustainability and scale-up of the interventions by the Ministry of Health and Ghana Health Service after the exit of the funder. For instance, the total cost projections for the health sector by the Ministry of Health was about GH₵ 45.9 million (approximately US$ 14.3million) for the periods 2014 to 2017 as contained in the 2014–2017 Health Sector Medium Development Plan (HSMTDP) policy document [11]. Comparing these projections with the total cost expenditure of nearly GH₵ 44.0 million (approximately US$ 13.7million) on MEBCI during a similar timeframe, the majority of which was expended in only four of the country’s ten regions and focused primarily on new-born care, calls for broader stakeholder dialogues on how this could be sustained by the MoH/GHS and subsequently scaled-up to other regions of the country.

A review of literature on donor support for resource-poor settings in Africa show that many laudable initiatives often financially propelled by donor agencies could not be sustained by beneficiary governments and health systems because they are too expensive for health budgets to accommodate [12–15]. While recognizing the limitations of the assumptions made in the cost evaluation analyses presented in this report, they could offer a pathway for the MoH, GHS and its partners to explore for more cost-efficient ways to scale up and sustain MEBCI in the future.

The World Health Organization (WHO) is increasingly propagating self-sustaining interventions towards attaining the United Nations-led Sustainable Development Goal (SDG) 3 [16, 17]. Internationally acceptable yet locally sustainable initiatives are significant impetus for ensuring resilience in health systems towards attaining health sector goals and universal health coverage. Many African countries, including Ghana, are constrained by severe macro- and micro-economic stability challenges which have the potential of reversing gains already made in the health sector, particularly in new-born care, if available health resources are not efficiently allocated and distributed [6, 7]. Ghana, like many African countries, continue to spend less than 15% of government expenditure on health. In light this, there is need for tailored-made interventions that suit the local conditions of communities if sustainability and scalability are to be realized.

It is however imperative to emphasize that this evaluation does not seek to conclude the new-born care interventions are expensive per se relative to similar interventions in Ghana or elsewhere. It would be premature to draw this conclusion because the evaluators did not do comparative cost analysis within country or with other countries where similar interventions were implemented. Instead, what this study sought to do is present various scenarios of differences in cost per capita in respect of economies of scale and potential areas of cost savings (see Fig. 6) in future new-born care interventions. Likewise, available studies on cost of new-born care adopted significantly different designs and cost analysis approaches which do not make these studies comparable neither can they form the basis for advancing the argument on whether or not the cumulative and per capita costs are relatively high or low. In view of this, future researchers are encouraged to devote some time to comprehensive systematic review of cost data on previous interventions to inform the empirical basis for comparison on parameters of efficiency and value for money.

### Limitations

The authors acknowledge some limitations associated with the cost estimations. First, this focused on cost per capita by clinical and non-clinical staff other without disaggregating the per capita cost by the level of health facility mainly because new-born care interventions focus was more on the cadre of health staff and the geographic regional location of the staff than the level of health facility. Also, reporting of the cost figures by the various intervention districts and regions to the funding agent at the national level were not disaggregated according to level of health facility hence there was practically no disaggregated cost data by level of health facility. In view of these gaps in the available data future interventions design and implementation should have facility level financial reporting system to distil the cost per capita data from the facility level up to the national and regional levels to understand potential cost savings by level of health facility.

### Policy implications

1. First, it is recommended that strategies for scaling-up the MEBCI interventions to other regions be prioritized by relevant stakeholders. A scale-up of the MEBCI interventions based on lessons learnt will help provide a nationally representative empirical basis for adoption of the MEBCI approach towards improving new-born outcomes on Ghana.
2. Secondly, the Ghana Health Service and its partners should consider complementary alternatives to the large-scale off-site training system for health workers by leveraging emerging Information Communication Technology (ICT) solutions such as e-learning and m-learning which are relatively cost effective and perhaps more sustainable.
3. Thirdly, replication of the MEBCI approach at the health facility level to allow for satellite training of staff on the MEBCI standard protocols for new-born care through training of trainers (TOTs) will help reduce the cost of regional batch trainings.
4. Additionally, the Policy Planning Monitoring and Evaluation (PPME) unit of the GHS and MoH should consider a joint scientific impact assessment of the MEBCI interventions on key new-born indicators. This impact assessment will provide evidence for scale-up of the MEBCI interventions.
5. Finally, there is need for renewed commitment to new-born care and related health services through efficient resource allocation. This commitment could be achieved by increasing government budget allocation to health with a statutory fund dedicated to new-born care in Ghana.

## Conclusion

Over 70% of the cost associated with the MEBCI intervention was on capacity building and related activities for over 4000 staff to enhance new-born care in the four intervention regions. Counterintuitively, it was observed that the region with the highest cumulative cost of training also recorded the least cost per capita. The average cost per capita for all staff (including non-clinical staff) was approximately US$ 982 compared to US$ 799 for the core clinical staff. MEBCI interventions had a wide coverage in terms of training for frontline healthcare providers albeit the associated cost figures may be potentially expensive for the local health system to sustain.

Innovative training options could be employed to help reduce cost and achieve substantial scale in reaching health care workers throughout the country. Ensuring reasonable coverage and affordability of mobile data services in the areas where the health providers work would be a prerequisite to the success of this approach. Moreover, distilling which components of the training need to be in-person versus which one can be offered online would make this scenario more feasible.

## Data Availability

There are no restrictions to data and materials used in this manuscript and the project Principal Investigator, Dr. Nana A. Y. Twum-Danso, should be contacted on matters of data accessibility.

## Abbreviations

ECEB: Essential Care for Every Baby
GDHS: Ghana Demographic and Health Survey
GHS: Ghana Health Service
GoG: Government of Ghana
GSS: Ghana Statistical Service
HBB: Helping Babies Breathe
HSMTDP: Health Sector Medium Development Plan
ICT: Information Communication Technology
IP: Infection Prevention
KMC: Kangaroo Mother Care
MEBCI: Making Every Baby Count Initiative
MoH: Ministry of Health
NDMW: National Daily Minimum Wage
RHDs: Regional Health Directorates
RHs: Regional Hospitals
SDGs: Sustainable Development Goals
UK: United Kingdom
UN: United Nations
WHO: World Health Organization

## Footnotes

1 There were no readily available uniform US$ equivalence of all recorded expenditures in the four regions hence, the US$ equivalence as quoted in this report are based on the current prevailing exchange rate of 1 US$ = 4.45 GHC. US$ 4.45 Source: www.oanda.com/currency/converter Accessed on 27/09/2018. Accessed on 24/09/2018

2 2014 end of year exchange rate of 1 US$ = GHS 3.21. Source: www.oanda.com/currency/converter Accessed on 27/09/2018

3 2017 end of year exchange rate of 1 US$ = GHS 4.57. Source: www.oanda.com/currency/converter Accessed on 27/09/2018
